# Radical shift in the genetic composition of New England chicory populations

**DOI:** 10.1111/1365-2745.13968

**Published:** 2022-08-07

**Authors:** Tomáš Závada, Rondy J. Malik, Lisa Mazumder, Rick V. Kesseli

**Affiliations:** ^1^ Sterling College Craftsbury Common Vermont USA; ^2^ Biology Department University of Massachusetts Boston Boston Massachusetts USA; ^3^ Department of Ecology and Evolutionary Biology, Kansas Biological Survey Lawrence Kansas USA

**Keywords:** Asa Gray, *Cichorium intybus*, cpDNA, herbarium specimens, historical records, invasion biology, microsatellites, New England, population genetics

## Abstract

1. Human activities have been altering the flora and fauna across the planet. Distributions and the diversity of species, and the phenotypes of individuals in those species are changing. New England with its rapidly changing human demographics is an ideal place to investigate these temporal changes in the habitat. The flora of New England consists of both native and nonnative species. Non‐indigenous plant species have been introduced since the first Europeans arrived in North America in the 15th century. *Cichorium intybus* (chicory), native to Eurasia, was first recorded in North America in 1774. Subsequently, chicory spread and became naturalized throughout much of the continent.
2. In this study, we used chloroplast DNA sequences and 12 microsatellite nuclear markers to assess the temporal genetic changes in New England populations of chicory. We analysed 84 herbarium specimens and 18 contemporary extant populations (228 individuals in total).
3. Three chloroplast DNA haplotypes were detected and all were present in New England prior to 1890; however, Hap3 was rare prior to the 1950s. The nuclear DNA markers showed a major shift in the genetic diversity and composition, with all historical herbarium collections belonging to a single genetic cluster and 16 out of 18 contemporary chicory populations belonging to different genetic clusters. This change occurred regionally and also on a local scale with contemporary populations being very different from herbarium specimens collected previously in the corresponding localities.
4. *Synthesis*. Our results indicate that the genetic diversity and structure of *Cichorium intybus* populations have changed substantially since the founding populations in New England. These changes may have contributed to the success of this nonnative species and helped to fuel its rapid expansion and adaptation to the changing landscapes in both New England and the rest of North America.

## Graphical abstract

Populations of chicory today, are different from those that first colonized New England. All herbarium specimens, including one by Asa Gray, collected between 1848 and 1970, form a genetic group, distinct from nearly all contemporary collections. This shift in genetic composition likely reflect dispersal and selection to changing land use patterns that are part of New England history.

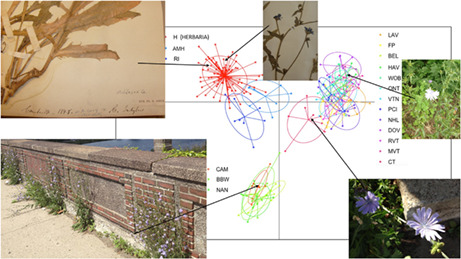

## 1 INTRODUCTION

The biodiversity on Earth has changed dramatically in the last 500 years largely due to activities of humans, with land use changes, biotic invasions, pollution and climate change being the major direct drivers (Daru et al., 2021; Pereira et al., 2012; Sala et al., 2000). Further, the impacts of these drivers decreasing biodiversity are severe (Tilman et al., 2014) and the prospects for averting this trend of global homogenization have not lessened despite our intensifying interest (Butchart et al., 2010; Pyšek et al., 2020). All of these drivers have contributed to changing biodiversity within New England in the USA, a region with a long history of well‐surveyed and documented flora.

The current flora of New England consists of both native and non‐native species. Non‐indigenous plants have been introduced since the first Europeans arrived in North America in the 15th century. Mehrhoff (2000) estimates that of the approximately 3000 vascular plants that comprise New England's flora, about 1000 are non‐native plant species and 200 are invasive. Many of these non‐native species were deliberately introduced and grown for food, forage, herbs and medicine. Through various modes of dispersal, these non‐native plants would often escape from cultivated fields and gardens.

*Cichorium intybus L*. (chicory), native to Eurasia, is an obligate outcrossing short lived perennial and a minor vegetable crop and a weed throughout much of North America. Chicory was introduced from Europe primarily as a semi‐domesticated species, arriving during the 1770s (Závada et al., 2017). The plant had a wide array of uses: a medicinal treatment for digestive ailments, a leaf and root crop, an ornamental, and sheep and horse fodder. While the first documented American sowing of chicory was by Thomas Jefferson, in 1774, with seeds obtained from George Washington (Looney, 2004), there have been several subsequent introductions from different sources (Závada et al., 2017). Governor James Bowdoin of Massachusetts brought chicory to New England and had it planted in his fields to feed sheep in 1785. The Rafinesque's (1811) list of more than 300 species had already considered chicory as common alongside roads and near gardens. The New England sheep industry peaked in 1840s, resulting in a wide introduction of chicory around the newly established sheep farms (Kains, 1898).

Several studies have examined the changes in floral and fruiting phenology over the past couple hundred years (Miller et al., 2021; Panchen et al., 2012; Wolkovich et al., 2013). Henry David Thoreau's notes were used to discern these patterns in New England, and those data have been linked to climate change (Willis et al., 2008; Willis et al., 2010). Chicory, mentioned in Thoreau's *Journal*, apparently spread quickly and became naturalized over the past 200 years in New England and across North America, but are the plants we see today, genetically the same as those seen by Thoreau, or have these populations shifted with time?

Herbarium specimens are valuable sources of information for plant taxonomic studies but also for the study of plant introductions and changes in phenology and distribution (Davis et al., 2015; Meineke et al., 2019). Further, novel uses to assess phenotypic and genotypic change have been rising in the last decade (Heberling & Isaac, 2017). In our study, we extracted DNA from herbarium specimens collected as early as 1848 by the first full‐time professor of botany at Harvard University, Asa Gray (Table S1—herbaria specimen GH00587961). We assayed the genetic make‐up of historical accessions and of extant populations from the same sites, to characterize any temporal shifts in the genetic composition of New England chicory over the last 170‐year period.

## 2 MATERIALS AND METHODS

### 2.1 Plant material

Leaves of historical *Cichorium intybus* accessions were obtained from five New England herbaria—University of Massachusetts Amherst Herbarium (MASS), University of Massachusetts Boston Herbarium (UMB), Harvard University Herbaria (ECON, GH and NEBC collections), The Hodgdon Herbarium at University of New Hampshire (NHA), and Nantucket Maria Mitchell Association (NMMA) herbarium (Accessed through the Consortium of Northeastern Herbaria web site, www.neherbaria.org). We sampled 84 herbarium specimens and the collection dates ranged from 1848 (Asa Grey's mounting sheet) to 2004 (Table S1). Leaf tissue from each herbarium specimen was transferred from its mounting sheet with clean forceps and placed into an individual envelope for DNA extraction. All vouchers except for UMB accessions have been digitized by the Consortium of Northeast Herbaria. We chose herbaria specimens with well‐described collection site information (town's neighbourhood and in several cases the street name) and were able to locate 18 extant, contemporary populations in approximately the same sites (Table 1). Leaves from eight individuals at each of the 18 matching extant populations were collected and kept at 4°C.

**TABLE 1:** Contemporary extant chicory populations in New England, USA

Population	*N*	Location	GIS	Ho	He	F	cpDNA	Herbaria cpDNA
CAM	8	JFK St, Cambridge MA	42.37362, −71.10973	0.5104	0.5125	0.0041	1	1 (1848)
LAV	8	Lexington Ave, Cambridge MA	42.38068, −71.14128	0.6136	0.6327	0.0301	2	2 (1913)
FP	8	Fresh Pond, Cambridge MA	42.38566, −71.14931	0.6136	0.6144	0.0008	2	2 (1940)
BEL	8	Belmont, MA	42.39564, −71.17761	0.7083	0.5651	−0.254	2	2 (1902)
BBW	8	Beaver Brook Res, Waltham MA	42.3929, −71.19717	0.6	0.5583	−0.0746	2	2 (1919)
NAN	8	Nantasket beach, Hull MA	42.3048, −70.90662	0.5972	0.5954	−0.0031	2	2 (1890)
HAV	8	Haverhill, MA	42.7762, −71.07728	0.5273	0.6897	0.2355	**3,2**	**1 (1935)**
WOB	8	Woburn, MA	42.47926, −71.15228	0.5568	0.4977	−0.1187	2	2 (1880)
ONT	8	Orange St, Nantucket MA	41.2762, −70.09453	0.5796	0.7129	0.1870	**3,1**	**1 (1889)**
VTN	8	Vestal St, Nantucket MA	41.28008, −70.1095	0.6591	0.6803	0.0312	**3,1**	**2 (1928)**
AMH	8	Amherst, MA	42.367, −72.517	0.5729	0.6444	0.1110	1	1 (1868)
RI	8	Middletown, RI	41.52182, −71.28345	0.4167	0.5708	0.2701	1,2	1 (1908)
PCI	8	Portland, ME	43.66147, −70.25533	0.6039	0.6545	0.0773	**2,1**	**1 (1900)**
NHL	8	Durham, NH	43.13895, −70.93703	0.5114	0.5689	0.1012	1	1 (1941)
DOV	8	Dover, NH	43.19786, −70.87367	0.6542	0.5679	−0.1521	**3**	**2 (1931)**
RVT	8	Rutland, VT	43.61062, −72.97261	0.5747	0.6502	0.1161	1	1 (1908)
MVT	8	East Middlebury, VT	43.97339, −73.10623	0.5893	0.6158	0.0431	**3,1**	**1 (1908)**
CT	8	Storrs, CT	41.80843, −72.24952	0.6136	0.6167	0.0049	**1**	**3 (1969)**

Abbreviations: F, inbreeding coefficient; He, average expected heterozygosity; Ho, average observed heterozygosity; *N*, sample size.

### 2.2 DNA extraction, PCR and genotyping

FastDNA extraction kit (MP Biomedicals) was used for DNA extractions of both the 84 dried herbarium specimen tissue and 144 fresh samples from the 18 extant populations according to manufacturer's protocol. We used 12 microsatellite nuclear markers (Závada et al., 2017), and one intergenic spacer *trnL*‐*trnF* chloroplast marker (Taberlet et al., 1991) to score the genotypes of the 228 individuals of this study.

The 25 μl polymerase chain reactions (PCRs) to amplify the selected markers contained: 5 μl DNA (20–100 ng), 2.5 μl of 2.5 mM MgCl_2_, 2.5 μl of 2 mM deoxynucleotides (dNTPs), 2.5 μl of 10× reaction buffer, 1 μl of each primer (10 mM), 0.2 μl of *Taq* polymerase (5 units/μl) (New England Biolabs, Ipswich, Massachusetts) and 10.3 μl H_2_O. The thermocycler (MJ Research PTC‐100, Waltham, Massachusetts) conditions were 95°C for 5 min, 35 cycles of 95°C for 30 s, annealing temperature 52°C for 30 s, 72°C for 45 s, and a final extension of 72°C for 5 min. We followed a modified amplification protocol for herbaria specimens with the KAPA3G Plant PCR Kit—Mix B (Sigma Aldrich, St. Louis, Missouri), as optimized in Schori et al. (2013).

### 2.3 Data analysis

Chloroplast DNA (cpDNA) fragments were sequenced and PCR products targeting microsatellite regions were assayed on the 3100‐*Avant* sequencer (Applied Biosystems). We used the program Sequencher 4.9 (http://genecodes.com/) for chloroplast DNA sequence editing and alignment. Peak Scanner software was employed for microsatellite fragment length scoring (Applied Biosystems). Peaks were assigned numbers by Peak Scanner based on the 400HD Rox size ladder that approximated the length of amplicons. Each individual peak size was confirmed visually. The observed (H_o_) and the expected (H_e_) heterozygosity, inbreeding coefficient (*F*) and the analysis of molecular variance (AMOVA) were calculated using Arlequin v. 3.5 (Excoffier et al., 2005). Significance of *Φ*_ST_ values was determined via the maximum number of permutations in Arlequin 3.5. Allelic analyses were calculated using a rarefaction method in HP‐Rare (Kalinowski, 2005). Chloroplast DNA haplotype maps were constructed using GPS visualizer (http://www.gpsvisualizer.com/).

A Bayesian clustering approach, program STRUCTURE v. 2.3.4 (Falush et al., 2003; Pritchard et al., 2000), was applied to evaluate the ancestry and the genetic composition of *Cichorium intybus* accessions. All sampled individuals were allowed to be products of admixture, and we used prior information about the population origin. The length of burn‐in period was set to 200,000 iterations, and the number of Markov Chain Monte Carlo (MCMC) steps after burn‐in was 1,000,000. We conducted five independent runs with a partial data set (84 herbarium specimens, with *K* set from 1 to 7), and with a complete data set (228 individuals) with K set from 1 to 10, with 10 iterations for each K in each independent run. STRUCTURE results were run through STRUCTURE HARVESTER v. 0.6.93 (Earl & VonHoldt, 2012) to calculate Δ*K* for each value of *K* according to Evanno et al. (2005) and plot the logarithm probability of each *K*. The STRUCTURE HARVESTER output data was permuted with CLUMPP v. 1.1.2 (Jakobsson & Rosenberg, 2007). The final visualization of genetic data was plotted with DISTRUCT v. 1.1 (Rosenberg, 2004). We explored the STRUCTURE analyses with individual loci and with each locus removed from the full analysis to identify possible locus specific artefacts. For visual clarity of individual population changes, we conducted analyses and displayed the results with the 31 herbarium specimens for which we found contemporary populations within their herbarium historical group and also repeated with their physical contemporary site groups. We have run the analyses multiple times and inclusion of these samples in two positions within the analyses does not change any of the relationships of the populations or the genetic composition of the individuals.

To corroborate results from the STRUCTURE analyses, a model‐free discriminant analysis of principal components (DAPC) was performed for the microsatellite loci data in the R package adegenet version 2.1.1 (Jombart, 2008; Jombart & Ahmed, 2011). Cross‐validation and determination of accurate number of principal components (PCs) was performed according to the tutorial in https://grunwaldlab.github.io/Population_Genetics_in_R/DAPC.html. Population structure was inferred for groupings of samples belonging to ‘H’‐herbarium against all other non‐herbarium contemporary samples. To explore the influence of single loci on population structure, the DAPC analysis was also performed with each of the microsatellite loci removed. The analyses were repeated with samples categorized into herbarium samples, herbarium samples from the same locations as extant populations, and contemporary samples to test whether the herbarium samples from extant population sites grouped more closely with all other herbaria samples or their corresponding contemporary population.

## 3 RESULTS

### 3.1 Chloroplast markers

**FIGURE 1:**
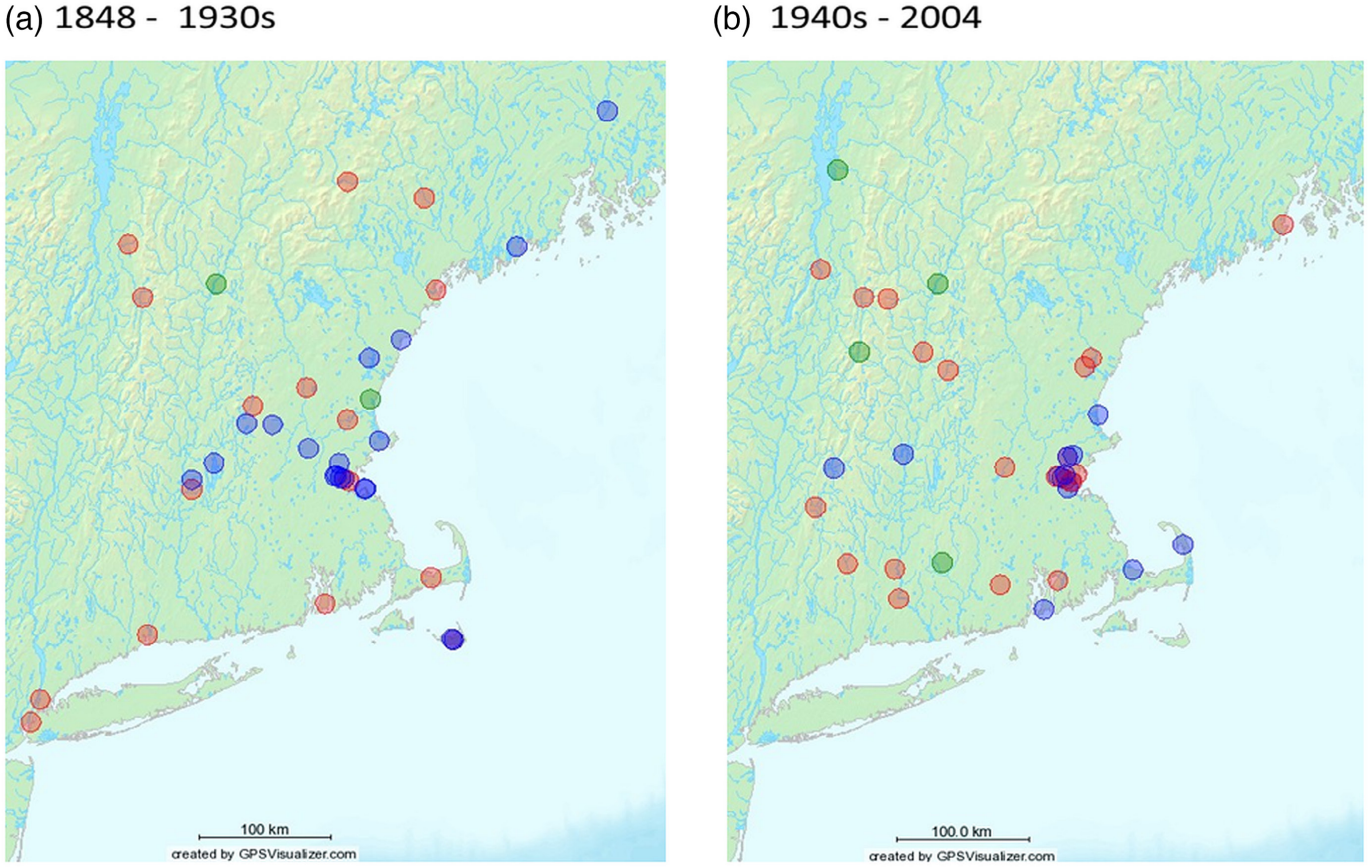
Geographical distribution of cpDNA haplotypes in herbaria specimens: (a) 1848–1939 and (b) 1940–2004. Hap 1—red, Hap 2—blue, Hap 3—green.

**FIGURE 2:**
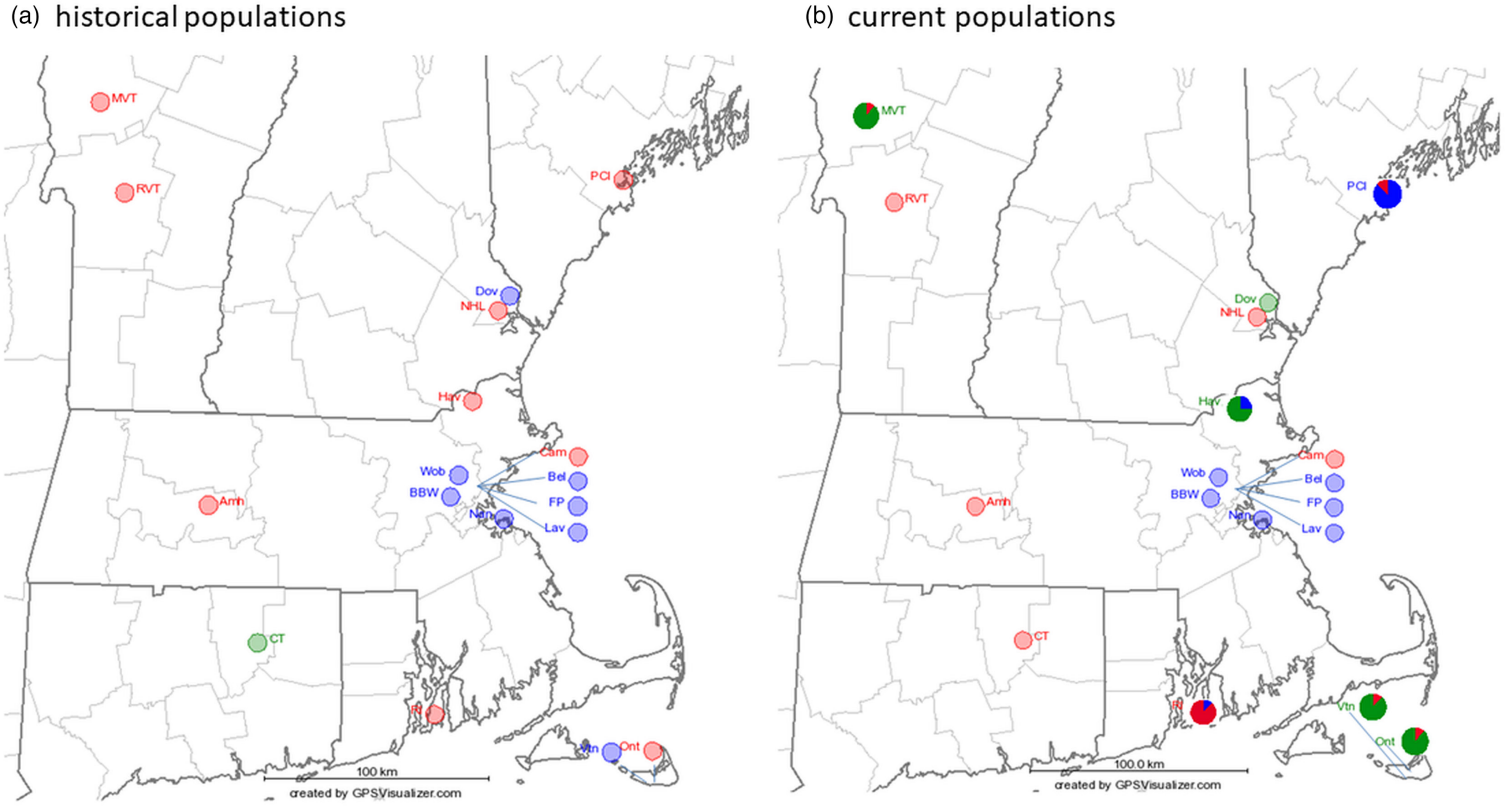
Geographical distribution of cpDNA haplotypes in chicory specimens: (a) herbaria (b) contemporary populations; Hap 1—red, Hap 2—blue, Hap 3—green.

All samples from contemporary extant populations and 84 herbarium specimens were sequenced at the *trnL*‐*trnF* locus. We could not obtain high quality sequence reads from four herbaria DNAs (H13, AM10, AM9, AM19). We detected three cpDNA haplotypes (Table 2) that were 702–705 bp long. The GenBank accession numbers for these three haplotypes are OL519592, OL519593 and OL519594. All three haplotypes were present in New England prior to 1890 (Figure 1) and persisted in the environment until present (Figures 1 and 2). However, there appear to be subtle temporal changes in the haplotype frequency. Within all herbarium collections, Hap1 and Hap2 were detected in the earliest collections and represented 49% and 43% of the samples, respectively. Hap3 appeared slightly later (1890) but represented only 8% of the full set of 80 samples and was restricted to New Hampshire collections until the 1960s. Within the smaller subset of 31 herbarium samples chosen because they resided at the 18 contemporary population sites, these frequencies were similar with 56%, 40% and 3%, respectively. However, the frequency of Hap3 was higher in the 144 samples of the 18 contemporary populations, with frequencies of the haplotypes being 35%, 40% and 24%, respectively. Six out of 18 extant contemporary populations (HAV, ONT, VTN, RI, PCI and MVT) were polymorphic for cpDNA haplotypes. In four populations (HAV, VTN, DOV and CT), the contemporary sample did not carry the cpDNA haplotype previously detected in the matching herbarium specimen and both HAV and VTN had all three haplotypes in their history (Table 1).

**TABLE 2:** Haplotype assignment based on cpDNA sequences

cpDNA haplotype	GenBank accession	SNP/ INDEL positions	Populations
		*67–69*216 *570*666*	
Hap1	OL519592	* AGC * G * G * T *	Cam, Amh, RI, NHL, RVT, CT, Ont, Vtn, PCI, MVT
Hap2	OL519593	* ‐‐‐ * A * G * T *	Lav, FP, Bel, BBW, Nan, Wob, PCI, Hav, RI,
Hap3	OL519594	* AGC * G * C * T *	Hav, Ont, Vtn, Dov, MVT

### 3.2 Nuclear markers

Twelve assayed microsatellite loci were polymorphic (Závada et al., 2022; https://doi.org/10.5281/zenodo.6820061), and markers amplified in all 228 individuals. We have detected 153 alleles with 96 alleles in historical, herbarium population and an increase to 140 alleles in contemporary populations (Table 3). The historical collection had 13 alleles not found in the contemporary collection, but the contemporary collection had 57 unique alleles not found in the historical collection. The number of alleles per locus ranged from 7 to 27. The average number of observed alleles over 12 loci was higher in contemporary populations (11.7) than the historical, herbaria group (8.0). These allelic changes were remarkable for some loci. For example, Locus 1385 showed 7 alleles in the historical collection and 23 in the contemporary collection (Table 3). The most common allele in the historical collection had a frequency of 0.73 while that same allele had a frequency of less than 0.02 in the contemporary collection. The AMOVA (Table 3) revealed significant population structure at all levels and that 11.7% of the variance is partition between historical and contemporary collections.

**TABLE 3:** Locus‐specific population genetics statistics and AMOVA examining herbarium and contemporary groups of individuals

Locus	All samples	Historical/herbarium		Contemporary	
	No. of alleles	No. of alleles	Private alleles	No. of alleles	Private alleles
5291	16	14	2	14	2
3984	11	8	2	9	3
5055	7	4	0	7	3
12,770	12	8	0	12	4
6865	9	8	1	8	1
1385	24	7	1	23	17
11,019	27	8	0	27	19
13,676	11	9	2	9	2
2050	9	9	2	7	0
3899	8	7	1	7	1
7179	11	7	1	10	4
8271	8	7	1	7	1
Mean	12.8	8.0	1.08	11.7	4.75
Total	153	96	13	140	57

Sources of variation	*df*	Sum of squares	Variance components	Percent
Between groups	1	262.18	1.18	11.7*
Within groups	226	2451.48	1.89	18.6*
Within individuals	228	1613.37	7.08	69.7*
Total	455	4327.03	10.15	100

* *p* < 0.001.

**FIGURE 3:**
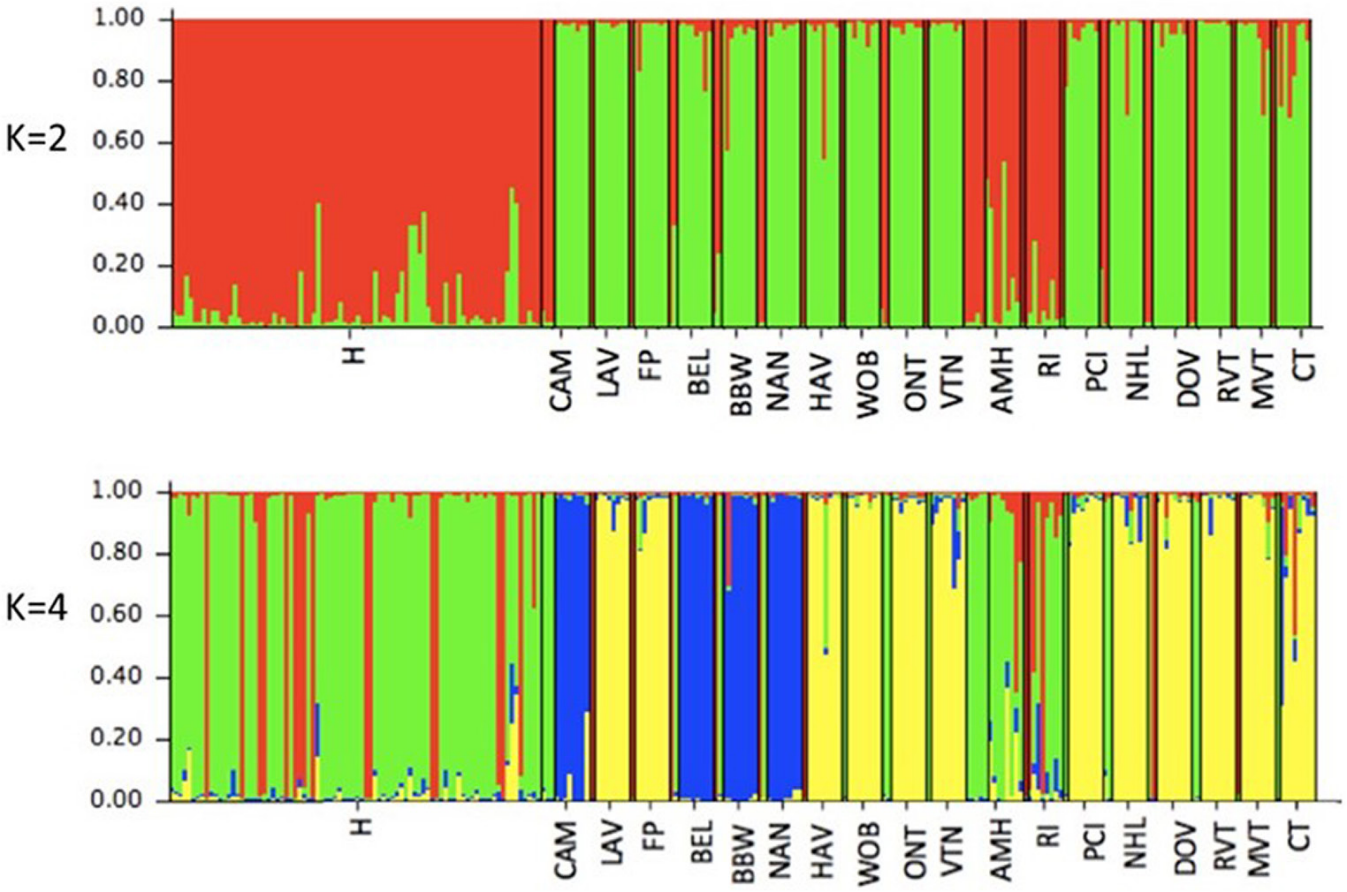
STRUCTURE analyses and inference of population structure in 84 herbarium chicory specimens and 18 extant contemporary chicory populations (total of 228 specimens), assuming *K* = 2 and *K* = 4. Herbarium specimens (H) are shown chronologically on the left, from 1848 through 2004, as listed in Table S1 and extant contemporary sites are described in Table 1 and labelled by site. The 31 historical herbarium specimens, which resided at the 18 contemporary population sites are shown twice in the figure; first among the 84 herbarium specimens and, second separated by thin black divider lines and preceding each of the corresponding contemporary population.

Using the 12 microsatellite markers, we analysed the population structure of these samples in several ways. We conducted a partial STRUCTURE analysis for 84 herbarium specimens; the number of clusters (*K*) was varied from one to seven. The highest likelihood in the partial analysis was obtained when *K* was set to four. The maximal Δ*K* occurred at *K* = 2 using the method of Evanno et al. (2005), with a slightly lesser peak at *K* = 4. The plot with the mean logarithm probability for each *K* shows the asymptote is reached at *K* = 4 (Figure S1). The STRUCTURE analysis for the full data set of 84 herbarium specimens and 144 individuals from the 18 contemporary populations was also conducted with *K* = 1 to 10 clusters. The maximal Δ*K* occurred at *K* = 2, with a smaller peak at *K* = 4. The mean logarithm probabilities approach the asymptote at *K* = 4 and beyond (Figure S2). We report both scenarios for *K* = 2 and *K* = 4 (Figure 3). All 84 herbarium samples belong predominantly to a single genetic group (identified as red for *K* = 2) or two groups (red and green for *K* = 4) while all samples in 16 of the 18 contemporary populations belong predominantly to different genetic groups (identified as green for *K* = 2 and yellow and blue for *K* = 4). Note, for visual clarity of direct population comparisons, we have repeated the analyses and display the 31 herbarium samples which historically resided at the sites of these 18 contemporary populations, both among the herbarium collections, and at the start of, and adjacent to, their corresponding contemporary population. Remarkably, the analyses suggest that old herbarium specimens from throughout the region are similar to each other and often distinct from the contemporary populations in the same locations. Exploration of the STRUCTURE analyses did not reveal any aberrant loci or possible artefacts that could create these patterns (data not shown).

**FIGURE 4:**
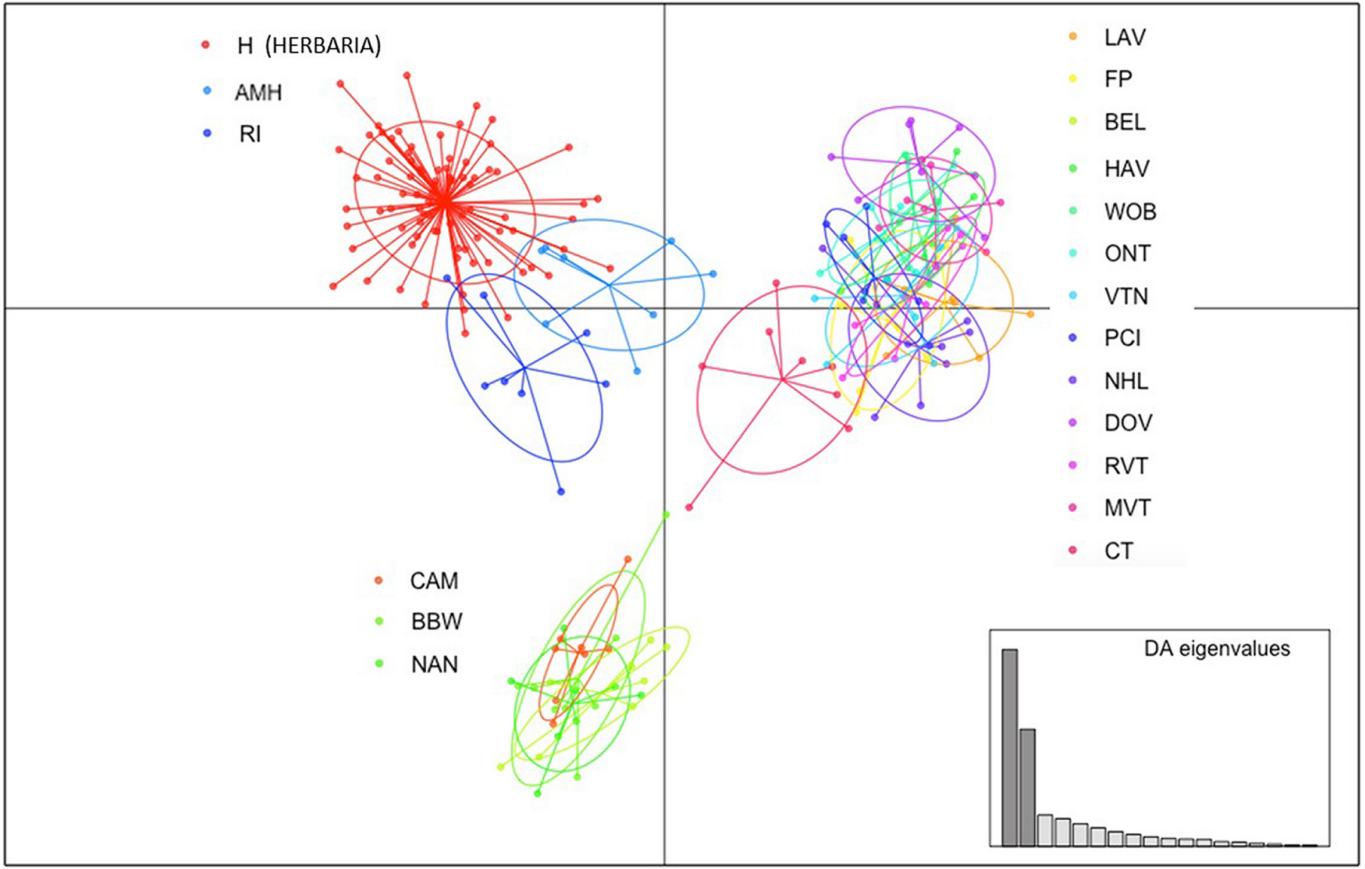
Discriminant analysis of principle component (DAPC) and inference of population structure in 84 herbarium chicory specimens and 18 extant contemporary chicory populations (total of 228 specimens). Each point represents one sample and the colours represent the 84 herbarium specimens (red) or the 8 individuals from each of the 18 contemporary sites. Ovals surrounding each population denote the 95% confidence intervals.

The 84 herbarium samples also clearly separated from contemporary samples in the results of the DAPC analyses (Figure 4). Herbarium samples overlapped slightly with contemporary extant populations belonging to RI and AMH locations, a pattern also seen with the STRUCTURE analysis, but had no overlap with any of the other contemporary samples. The 31 herbaria samples that had once resided at the 18 extant contemporary sites also overlapped with the group of remaining herbarium samples as a whole and not with their corresponding extant populations. In all exploratory analyses with DAPC examining the impacts of single loci, the herbarium samples always consistently separated from non‐herbarium, contemporary samples.

## 4 DISCUSSION

Our study is the first temporal genetic analysis of non‐indigenous chicory species in North America. Focusing on New England, we utilized a collection of 84 herbarium specimens spanning 170 years. From this collection, we identified 31 specimens that located to 18 sites across New England and were able to find contemporary extant populations of chicory in all of these sites. The herbaria and extant collections from 18 populations were used to infer genetic structure of historical and contemporary populations. Combining the genetic and temporal data, we observed a dramatic shift in the composition of New England chicory populations over time.

We detected three cpDNA haplotypes in New England, consistent with an earlier study of North American collections (Závada et al., 2017). The comparison of modern and historical samples showed that the three haplotypes were present by the 1890s but Hap3 was relatively rare prior to the 1950s (Figures 1 and 2). Ten of the 18 contemporary populations revealed only one haplotype, which matched the older herbarium collections. The eight other populations either had additional haplotypes beyond the one detected in the herbarium collection (four populations) or switched and carried different haplotypes from the original herbarium collection (four populations). Hap3, which was rare in the original herbarium collections, was the majority haplotype in 5 of 18 contemporary populations. These data suggest multiple introductions via seed and a shift in the genetic structure of populations.

Allelic analyses of microsatellite data revealed enhanced genetic diversity in the contemporary populations of chicory—we detected 57 unique alleles in the contemporary populations that were not present in the historical herbaria collections, but only 13 unique alleles in the historical data set (Table 3). While the contemporary collection was larger (144 total samples), we might not expect it to be more diverse; it was derived from 18 locations collected in one season while the 84 herbarium samples were collected from many more locations over 170 years. This increase in allelic diversity within the contemporary collection could have been fueled by several subsequent introductions enriching the gene pool, and possibly the accumulation of new mutations or shifting selection pressures (Heilbron et al., 2014; Simberloff, 2009). Most dramatically, the overall structure of these contemporary populations changed with 16 of 18 populations showing a different genetic history from the older herbarium collections.

Several factors may have contributed to this surprising outcome; recent introductions, anthropogenic seed dispersal and gene flow, and changing landscapes associated with agricultural activities and climate change. The New England landscape has been dramatically transformed multiple times, since the colonization by Europeans. Early deforestation for agriculture, sheep farming and lumber with the accompanying introduction of many non‐native species such as chicory. Subsequently, both agriculture and the lumber industry largely moved to the Midwest starting in the latter half of the 19th century, and fields and pastures in New England became abandoned. Sheep farming in particular reached its pinnacle in New England during the 1840s, leading to large populations of chicory as a fodder crop. These agricultural and disturbed environments proved to be excellent habitats for chicory. Chicory abundance was not reported to be problematic during 19th century, but this may have changed, as chicory began to spread across the continental United States. After more than 100 years on the continent, farmers in the early 1900s began to call for the control and eradication of chicory (Hansen, 1920). Although previous studies showed that high genetic diversity is not crucial for colonizing and persisting in a new environment (Ward et al., 2008), non‐indigenous species with complex genetic structures and exposed to changing environments and selective pressures, might adapt faster.

Globalization and anthropogenic activity have reshaped the biodiversity and the evolutionary path and success of many plant species. Native population distributions are shifting, and weeds and invasive species have successfully colonized nearly every human‐disturbed environment on the planet. In addition, phenotypic changes during historical times, such as flowering times, have been increasingly reported in plant populations. These shifts provide an opportunity and need to investigate landscape genetics and evolutionary changes, through time and space to tease apart the roles of genetic adaptation, phenotypic plasticity and stochastic processes to these dynamic systems. Employing contemporary and historical collections dating back to 1848, we have provided a more complete understanding of the population history of the nonindigenous weed chicory in North America. Our data indicate that cryptic genetic changes underlie the success of chicory as it has adapted to a rapidly changing landscape over the last 170 years.

## AUTHOR CONTRIBUTIONS

Rick V. Kesseli and Tomáš Závada designed the experiment; Tomáš Závada and Rondy J. Malik carried out the field and lab work; Data analysis was done by Tomáš Závada, Lisa Mazumder and Rick V. Kesseli; Tomáš Závada and Rick V. Kesseli wrote the manuscript and all authors contributed critically to drafts.

## CONFLICT OF INTEREST

None of the authors have a conflict of interest associated with this work.

### PEER REVIEW

The peer review history for this article is available at https://publons.com/publon/10.1111/1365‐2745.13968.

## Supporting information


Figure S1

Figure S2

Table S1

## Data Availability

Chloroplast DNA sequences of the *trnL‐trnF* intergenic spacer are available in GenBank (accessions OL519592, OL519593 and OL519594). Microsatellite data are available from Zenodo https://doi.org/10.5281/zenodo.6820061 (Závada et al., 2022).
