# Supplementary material for: Radical shift in the genetic composition of New England chicory populations
Source: J Ecol. 2022 Aug 7;111(2):391–9. doi: 10.1111/1365-2745.13968 (PMC10087836; doi:10.1111/1365-2745.13968)
Supplement: Supplementary file 1 — Figure S1 [file JEC-111-391-s002.docx]

**Supporting Information, Figure 1:** Plots of (A) Δ K, and (B) the log likelihood from the Structure analysis of the partial data set (84 individuals – herbarium specimens).

**A**

**B**
