# Supplementary material for: Radical shift in the genetic composition of New England chicory populations
Source: J Ecol. 2022 Aug 7;111(2):391–9. doi: 10.1111/1365-2745.13968 (PMC10087836; doi:10.1111/1365-2745.13968)
Supplement: Supplementary file 2 — Figure S2 [file JEC-111-391-s003.docx]

**Supporting Information, Figure 2:** Plots of (A) Δ K, and (B) the log likelihood from the Structure analysis of the complete data set (228 individuals).

**A**

**B**
