# Supplementary material for: Radical shift in the genetic composition of New England chicory populations
Source: J Ecol. 2022 Aug 7;111(2):391–9. doi: 10.1111/1365-2745.13968 (PMC10087836; doi:10.1111/1365-2745.13968)
Supplement: Supplementary file 3 — Table S1 [file JEC-111-391-s001.docx]

Supporting Information **Table 1:** Herbarium specimen collection

**ID Year Location Herbarium ID cpDNA**

**H6 1848 Cambridge, MA GH00587961 1**

AM28 1863 New Haven, CT MASS00349466 1

NH14 1863 Wells, ME NHA536388 2

AM29 1864 Inwood, NY MASS00349466 1

AM30 1866 New York, NY MASS00349466 1

**AM14 1868 Amherst, MA MASS00349450 1**

**NH3 1880* Woburn, MA NHA536690 2**

**MM1 1889 Orange St, Nantucket MA NMMA000349 1**

**H14 1890 Nantasket beach, Hull MA NEBC00535253 2**

H10 1890 Westford, MA GH00587987 2

NH10 1890* Hanover, NH NHA536699 3

**H7 1893 Cambridge, MA GH00587963 2**

NH13 1896 Orono, ME NHA536391 2

H15 1898 Bristol, ME NEBC00587912 2

H16 1898 Buckfield, ME NEBC00587907 1

**NH12 1900 Portland, ME NHA536387 1**

AM16 1902 Boston, MA MASS00349457 1

AM17 1902 Boston, MA MASS00349458 1

**H5 1902 Belmont, MA NEBC00587959 2**

NH6 1905 Manchester, NH NHA536701 1

**AM42 1908 Rutland, VT MASS00349480 1**

**H20 1908 Middletown, RI GH00535279 1**

**H21 1908 East Middlebury, VT GH00587946 1**

H2 1909 Essex, MA GH00587952 2

**MM2 1909 Nantucket, MA NMMA000347 2**

H13 1912 Milton, MA GH00535247

**H8 1913 Lexington Ave, Cambridge MA NEBC00587964 2**

NH7 1913 Peterborough, NH NHA536700 1

H19 1916 Hampton Falls, NH NEBC00587937 3

H17 1917 Mason, NH NEBC00587939 2

AM33 1918 Shelburne, NH MASS00349470 1

H18 1918 Rindge, NH NEBC00587943 2

**H4 1919 Beaver Brook Res, Waltham MA NEBC00312020 2**

H12 1919 Barnstable, MA NEBC00535255 1

**AM10 1928 Amherst, MA MASS00349444**

**AM12 1928 Amherst, MA MASS00349448 2**

**ID Year Location Herbarium ID cpDNA**

**MM3 1928 Vestal St, Nantucket MA NMMA000348 2**

AM7 1929 Millington, MA MASS00317212 2

AM1 1931 Pemberton, MA MASS00349456 2

**AM19 1931 Hull, MA MASS00349455**

AM9 1931 Southampton, MA MASS00349446

**NH8 1931 Dover, NH NHA536704 2**

**AM11 1932 Amherst, MA MASS00349445 2**

**AM13 1932 Amherst, MA MASS00349447 2**

**H3 1935 Haverhill, MA NEBC00587953 1**

**H1 1940 Fresh Pond, Cambridge MA NEBC00587960 2**

**NH5 1941 Durham, NH NHA536703 1**

**H9 1948 Cambridge , MA GH00587965 1**

NH11 1950 Hanover, NH NHA536709 3

AM5 1967 Mt Hope Bay, MA MASS00349439 1

**AM37 1967 North Rutland, VT MASS00349479 1**

AM39 1967 East Dorset, VT MASS00349476 3

AM43 1967 West Bridgewater, VT MASS00349480 1

**AM26 1969 Storrs, CT MASS00349467 3**

AM8 1970 Deerfield, MA MASS00349442 2

B1 1970 625 Huntington Av, Boston MA UMB1912 2

**B2 1970 Belmont st, Belmont MA UMB1913 2**

**B3 1970 Beaver st, Waltham MA UMB1914 1**

B4 1970 Revere, MA UMB1915 1

**NH9 1970 Dover, NH NHA536708 1**

AM32 1972 Marlow, NH MASS00349469 1

AM36 1972 Newport, RI MASS00349474 2

H11 1972 Roxbury, MA ECON00535242 2

AM2 1973 Wellfleet, MA MASS00349436 2

AM4 1973 Pittsfield, MA MASS00349438 2

AM25 1974 Torrington, CT MASS00349468 1

AM23 1974 Rockland, ME MASS00349464 1

AM38 1974 Chipman's point, VT MASS00349475 1

AM18 1975 Boston, MA MASS00349459 1

AM20 1975 Medford, Tufts Campus, MA MASS00349451 2

AM41 1975 Burlington, University of Vermont MASS00349478 3

**AM34 1976 Durham UNH, NH MASS00349471 1**

AM24 1977 Middletown, CT MASS00349452 1

**ID Year Location Herbarium ID cpDNA**

AM27 1977 Hartford, CT MASS00349463 1

AM22 1977 Middlesex, CT MASS00349465 1

AM35 1977 Kent County, RI MASS00349473 1

NH1 1977 Plum Island, MA NHA539661 2

NH2 1977 Reading, MA NHA536691 2

AM31 1980 Charlestown, NH MASS00349472 1

NH4 1988 Egremont, MA NHA536688 1

AM15 1991 Lancaster, MA MASS00349460 1

AM21 1993 Medford, MA MASS00349453 2

AM3 1994 Sandwich, MA MASS00349437 2

AM6 2004 Lynnfield, MA MASS00349440 2

**Origin**: ECON, GH and NEBC – Harvard University Herbaria collections, MASS - University of Massachusetts Amherst Herbarium, NHA - The Hodgdon Herbarium at University of New Hampshire, NMMA - Nantucket Maria Mitchell Association herbarium, UMB - University of Massachusetts Boston Herbarium The bold formatting indicates the 31 herbaria specimen used for the comparison with the 18 contemporary extant populations. Asterisks indicate missing collection date that was extrapolated based on the collector’s life span.
